# The use of free resources in a subscription-based digital library: a case study of the North Carolina AHEC Digital Library

**DOI:** 10.1186/1742-5581-3-9

**Published:** 2006-09-06

**Authors:** Mary Beth Schell

**Affiliations:** 1 UNC Chapel Hill Health Sciences Library/CB # 7585/Chapel Hill, NC/27599, USA

## Abstract

**Background:**

The North Carolina (NC) Area Health Education Center's (AHEC) Digital Library (ADL) is a web portal designed to meet the information needs of health professionals across the state by pulling together a set of resources from numerous different sources and linking a pool of users to only the resources for which they have eligibility. Although the ADL was designed with the primary purpose of linking health care professionals to a set of licensed resources, the ADL also contains a significant number of links to free resources. These resources are available to any ADL member logging into their ADL account and to guest visitors to the ADL. While there are regular assessments of the subscription resources in the ADL as to utility and frequency of use, up until this point there has been no systematic analysis of the use of the overall set of free resources. It was decided to undertake an examination of the usage of ADL free resources over a 6-month period to analyze the utility of these resources to both ADL members and guests.

**Methods:**

Each time a resource is accessed through the ADL, it is logged in a table. This study used a SQL query to pull every free resource accessed between November 1, 2005 and April 30, 2006. An additional query also pulled the user information for each free resource accessed. Once the queries of the database were complete, the results were imported into an Excel spreadsheet and analyzed using basic descriptive statistics.

**Results:**

The vast majority of resource use through the ADL is to licensed resources. There are 2056 free resource URLs in the ADL, to which 1351 were linked out, meaning there was at least one link out to 65% of the free resources. The single most popular free resource was PubMed with 4803 link outs or nearly 20% of the total link outs to free resources. The breakdown of free resource use by different use groups indicates that the highest percentage of use of free resources was by guests followed by institutional affiliates and AHEC Faculty/Staff. The next 3 highest user groups accessing free resources are: paid members, preceptors, and residents.

**Conclusion:**

The only free resource capturing a significant number of link outs is the free link to PubMed. This reflects the importance placed on traditional medical literature searching by the ADL clinical user base. Institutional affiliates access free resources through the ADL with the second highest frequency of all the user groups. Finally, in analyzing use of free resources, it is important to note the overall limitations of this survey. While link outs are excellent indicators of frequency of use they do not provide any information about the ultimate usefulness of the resource being accessed. Further studies would need to examine not only the quantitative use of resources, but also their qualitative importance to the user.

## Background

The North Carolina (NC) Area Health Education Center's (AHEC) Digital Library (ADL) is a web portal designed to meet the information needs of health professionals across the state. Its core mission is to support the biomedical information needs of those health professionals affiliated with the NC AHEC Program (faculty, staff, preceptors and medical residents) as well as other community health professionals including hospitals, clinics, and individual providers. The North Carolina AHEC program began in the early 1970s as part of a nation-wide effort to improve the training and retention of health care professionals. The ADL is a unique digital library that pulls together a set of resources from numerous different sources and links a pool of users to only the resources for which they have eligibility. The network of regional AHEC libraries across the state supports the purchase of resources for AHEC-affiliated users, including those hospitals directly affiliated with an AHEC as well as those hospitals which have elected to participate in the digital library at an institutional level. Individual health professionals without eligible institutional affiliations may purchase individual annual memberships in the digital library.

The resources and services of the ADL are selected by health sciences librarians and health professionals, and include electronic databases, full-text health sciences books and journals, and high quality free Internet resources. Although the ADL was designed with the primary purpose of linking health care professionals to a set of licensed resources, the ADL also contains a significant number of links to free resources. Of the 3861 URLs in the ADL, 2056 or approximately 53% are links to free resources. These resources can be accessed by anyone with an Internet connection simply by logging into the ADL as a guest. These resources are also available to any ADL member logging into their ADL account. While there are regular assessments of the subscription resources in the ADL as to utility and frequency of use, up until this point there has been no systematic analysis of the overall set of free resources. It was decided to undertake an examination of the usage of ADL free resources over a 6-month period to analyze the utility of these resources to both ADL members and guests. A literature review conducted through the database Library and Information Science Abstracts (LISA) using a combination of keywords and descriptors including: free resources, free electronic resources, electronic resources, web resources, library catalogs, portals and gateways resulted in sets of anywhere between 2 and 71 articles. Most of the articles were reviews of free web resources on topics ranging from theater to cytotechnology. Other articles included several on how to evaluate web based resources and how to catalog web resources. The closest match was an article on the growing use of search engines such as Google and Yahoo by undergraduate students. This brief literature review conducted as part of this study revealed articles that weren't close enough to the subject of this article to provide insights into the topic. A more in depth literature review should be conducted in subsequent studies of the use of free resources.

## Methodology

The setting for the study is the ADL which is a dynamic web site. The material studied were the free URLs accessed through the ADL. The Free Dictionary and the Wikipedia both define a dynamic web site at its most basic level as a "Web site that delivers custom content to the user." [1] These dynamic web pages are created on the ADL by pulling resources that are housed in an SQL database into an HTML page based on codes in the user profile and codes in the resources profile. Each time a resource is accessed through the ADL, it is logged in a table. This study used an SQL query to pull every free resource accessed between November 1, 2005 and April 30, 2006. An additional query also pulled the user information for each free resource accessed. Once the queries of the database were complete, the results were imported into an Excel spreadsheet and analyzed using basic descriptive statistics. This statistical analysis comprised the comprehensive information about the research object of this study which was the free resource use in the ADL.

Free resource use was examined based on both resources accessed and on the user groups accessing the resources. The first parameter viewed was the overall number of link outs to free resources. This included looking at the specific resources and the frequency with which they were accessed. A link out is defined as each time an ADL user clicks on a resource through the ADL interface and links out of the ADL site to that resource. The second parameter viewed was the resources accessed by user group and the frequency of use of specific resources and resource type by user group.

## Results

During the November 2005 – April 2006 timeframe, there were a total of 127,031 link outs to resources. Of the 127,031 total link outs, 24,015 were to free resources. This is 19% of the total link outs. When the guest users are removed from the analysis, there were a total of 107,602 link outs to resources by registered ADL members. Of those link outs, 15,712 were to free resources. This indicates that 15% of the link outs by the registered users are to free resources. It is important to note that since guests only have access to free resources, 100% of resource use by guests as measured by link outs is for free resources. Despite the fact that a slim majority of resources are free, the vast majority of resource use through the ADL is to licensed resources. This confirms data gathered from a user survey in 2004 where users placed primary value on the ADL as a source for licensed resources. It seems clear that while the free resources are used, that they are not the primary resources accessed by ADL licensed users.

There are 2056 free resource URLs in the ADL, to which 1351 were linked out, meaning there was at least one link out to 65% of the free resources. The single most popular free resource was PubMed with 4803 link outs or nearly 20% of the total link outs to free resources. PubMed's popularity as the most accessed free resource can be seen in the fact that nearly 20% of the total link outs to free resources are to PubMed. The second most popular resource, MedlinePlus, only has approximately 3% of the total link outs to free resources. PubMed also ranks highly among all resources, free and licensed together – accounting for 3% of the total link outs. Other than the University of North Carolina at Chapel Hill (UNC) e-journals listing, PubMed is the only free resource in the top 10 most frequently linked to URLs in the ADL.

**Figure 1:**
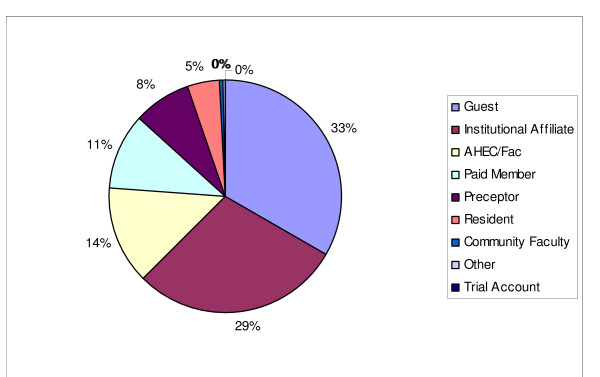
Free Resource Use by Account Type.

The breakdown of free resource use by different user groups indicates that the highest percentage of use of free resources was by guests followed by institutional affiliates and AHEC Faculty/Staff. The next 3 highest user groups are: paid members, preceptors, and residents. The final 3 groups registered less than 3% of the total use of free resources. These groups are: community faculty, other, and trial accounts. The breakdown of free resource use by different user groups is illustrated in figure 1. Due to the way that Excel rounded the numbers underlying the table, the figure indicates that the final 3 groups add up to 0% and give 100% of use to the Guests, Institutional Affiliates, AHEC Faculty, Paid Members, Preceptors, and Residents.

Not surprisingly the guests make up the largest group accessing free resources. The most likely reason for this is that guests only have access to the free resources. Although guests only make up 2% of total user link outs from the ADL, they make up 33% of the access to the free resources. While information on registered ADL members can be tracked, very little is known about the guest users.

**Figure 2:**
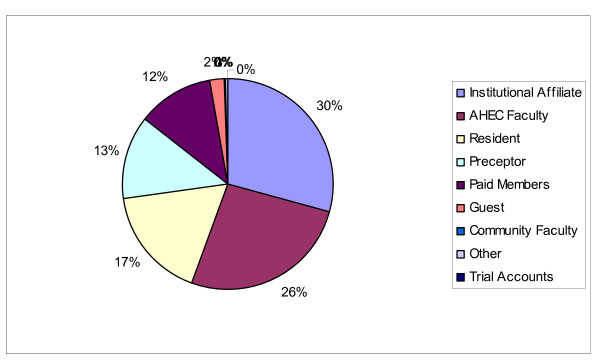
Total Link Outs to All Resources by Account Type.

It is also useful to look at free resource use without guest usage. When guests are removed from the equation, institutional users are the highest user group accessing free resources. Institutional affiliates are the largest active user group with 30% of total link outs during the period studied. The hypothesis that the largest free resource user groups will emerge from the largest overall user groups when excluding guests from the free resource analysis, gets mixed validation when examining actual ADL link outs as illustrated in Figure 2. When comparing overall link outs to link outs to free resources, 6 user groups align. The top 2 groups: Institutional Affiliates and AHEC faculty, and the 4^th ^highest link out group, the preceptors. The bottom 3 groups also aligned between their overall resource use and their use of free resources. There were 2 groups with some discrepancy between overall link outs and the link outs to free resources. The paid members are the 5^th ^highest overall link out group, but the 3^rd ^highest user group accessing free resources. Despite the difference in ranking, they have a similar use percentage with 12% of the overall resource use and a 16% use of the free resources use. The group with the largest discrepancy is the medical residents. They are the 3^rd ^highest overall link out group but only the 5^th ^highest group using free resources. The discrepancies with the medical residents are best highlighted in link out percentages: the overall link out compared to the link outs to free resources. The medical residents had 18% of the overall resource usage but only 7% of the use of free resources. Despite these 2 groups evincing discrepancies between overall resource usage and free resource usage, the overall usage patterns support the hypothesis that the largest free resource user groups will emerge from the largest overall user groups when excluding guests from the examination of the free resources.

In addition to examining overall free resource use by user group, we also examined the specific free resources accessed by each different user group. Tables 1, 2, 3, 4, 5 illustrate the top ten most frequently used resources overall and by user group. The tables provide the names of the resources and the total number of link outs during the November 2005 – April 2006 timeframe. Some highlights of those results include the fact that of the ten most frequently accessed free resources, five were to patient education resources. Of the five patient education resources accessed by institutional affiliates, three of the titles: Diseases, Conditions & Injuries; the MEDEM Library of Patient Education Information; and [MedlinePlus] Online Interactive Health Tutorials, account for more 50% of the total link outs for each title. For guests, 3 of the top 10 resources accessed most frequently were for nursing resources. They are the only group with any link outs to nursing resources in the top 10 most frequently accessed resources. AHEC Faculty/Staff accessed the EBM Center of Excellence for 40% of the link outs to that title. Another interesting note is that individual paid members accessed Dermatology resources at a much higher rate than any of the other groups.

**Table 1:** The top ten most frequently used free resources by all user groups: (resource name/link outs)

PubMed MEDLINE	4803
MedlinePlus: Health Information for the Public from NIH	636
Directory of Free Medical Journals	504
Drug Information from RxList	501
Diseases, Conditions & Injuries	428
Directory of Open Access Journals	378
eMedicine	348
EBM Education Center of Excellence	345
PubMed Central: Free Archive of Life Sciences Journals	305
Mayo Clinic Information for Patients	240

**Table 2:** Top ten most frequently used free resources by Guests.

PubMed MEDLINE	1012
Directory of Free Medical Journals	341
MedlinePlus: Health Information for the Public from NIH	295
Directory of Open Access Journals	264
Online Journal of Issues in Nursing	170
AHEC Connect	104
Internet Journal of Advanced Nursing Practice	102
Diseases, Conditions & Injuries	94
NC AHEC Statewide Continuing Education Calendar	91
Advance for Nurses	89

**Table 3:** Top ten most frequently used free resources by Institutional Affiliate Members.

PubMed MEDLINE	1071
Diseases, Conditions & Injuries	234
Drug Information from RxList	172
MedlinePlus: Health Information for the Public from NIH	156
MEDEM Library of Patient Education Information	129
eMedicine	102
Online Interactive Health Tutorials	83
EBM Education Center of Excellence	82
Directory of Free Medical Journals	70
PubMed Central: Free Archive of Life Sciences Journals	70

**Table 4:** Top ten most frequently used free resources by AHEC Faculty/Staff members.

PubMed MEDLINE	919
EBM Education Center of Excellence	138
Drug Information from RxList	93
eMedicine	77
PubMed Central: Free Archive of Life Sciences Journals	75
MedlinePlus: Health Information for the Public from NIH	73
UNC Chapel Hill Libraries Online Catalog	67
Directory of Free Medical Journals	51
Mayo Clinic Information for Patients	47
Directory of Open Access Journals	43

**Table 5:** Top ten most frequently used free resources by Paid Individual Members.

PubMed MEDLINE	1030
DermIS: Atlas of Dermatology	49
Drug Information from RxList	44
DermIS: Atlas of Pediatric Dermatology	40
National Guideline Clearinghouse	35
eMedicine	30
PubMed Central: Free Archive of Life Sciences Journals	30
Electronic Textbook of Dermatology	27
EBM Education Center of Excellence	26
Dermatlas.org	24

## Discussion

Since the free resources are the only resources available to the guests, it makes sense that they are the largest user group accessing these resources. Given that the ADL has always intended to meet the information needs of both registered members and guests, it would be useful at some point in the future to gather more information about the guest users either through a survey or some other mechanism. This would ensure that the free resources upon which the guests rely exclusively are meeting their information needs.

There are 4 user groups: guests, institutional affiliates, AHEC faculty/staff, and paid individual members whose top ten most frequently accessed free resources warrant a more in-depth discussion. As was stated earlier, all of the groups accessed the "free" PubMed link the most frequently out of all the free resources. Although there is some overlap among the resources in the top ten for each individual user group, the order of ranking varies among user groups. For example, the top 3 resources accessed by institutional affiliates were PubMed; Diseases, Conditions & Injuries (patient education materials); and Drug Information from Rx List, whereas the top 3 resources accessed by guests were PubMed, Directory of Free Medical Journals, and MedlinePlus (patient education). One possible reason that guests access a resource like the Directory of Free Medical Journals at a higher frequency than the other user groups is that they don't have access to the licensed resources and this resource could be one of their only access points into the medical literature.

Three of the top sites selected by guests were nursing specific: *Online Journal of Issues in Nursing, Internet Journal of Advanced Nursing Practice*, and *Advance for Nurses*. This suggests that a significant number of guest users of the ADL are nurses. A further study of guest users would be needed to verify this conclusion. Of all the user groups examined, guests are the only group accessing AHEConnect or the NC AHEC Statewide Continuing Education (CE) Calendar in their top 10 grouping of free resources. As a matter of fact, the guests link to AHEConnect for 52% of the total ADL link outs to AHEConnect and they link to the NC AHEC Statewide Continuing Education Calendar for 45% of all ADL link outs to this resource. When a further study of the guest users is conducted, examining why these two continuing education specific resources are so popular with this group would be useful.

Like all groups, institutional affiliates accessed the free PubMed the most often out of the free resources. This is interesting to note, since all of the institutional affiliates would also have access to Ovid's Medline with its links to full-text journals. When the institutional affiliates access the free PubMed, they have no direct access to full-text journals through their article searching. Despite the lack of access to full-text journals, the institutional affiliates accessed PubMed 15% of their total link outs to free resources. Further study will be needed to examine why the institutional affiliates are selecting PubMed over Ovid Medline. Another interesting aspect of the link outs to free resources by the institutional users is the preponderance of link outs to patient education resources. Further examination of whether or why institutional affiliates are finding certain specific patient education resources helpful would be interesting. It would help answer the question of whether the ADL should or could provide additional patient education resources to meet the needs of the institutional users.

It is interesting to note that of all the link outs to the Evidence Based Medicine (EBM) Center of Excellence, 40% of them came from AHEC faculty/staff. Of their top ten most often accessed resources, AHEC faculty/staff accessed EBM resources 9% of the time. A good follow-up question for this group would be one that would help understand why they access EBM resources at a higher frequency than other user groups.

The paid members diverge from the rest of the user groups by the preponderance of dermatology resources showing in the top ten most frequently accessed free resources. A possible explanation for this is that there could be a small group/single user who is a 'super user' of these materials and skews the usage numbers for the group as a whole. This theory could be tested by going back and re-running the statistics looking at individual link outs by user rather than an aggregate list by the user group.

## Conclusion

While predictably, use of free resources by guest users is heavy, the free resources in the ADL are not the primary resources visited by ADL members. The only free resource capturing a significant number of link outs is the free link to PubMed. This reflects the importance placed on traditional medical literature searching by the ADL clinical user base. The use of the "free" PubMed links with user groups who had access to a Medline database with full text links (via Ovid) is a question that will require further attention. Why do users who have access to literature searching with full text links select a resource that has very limited links to full text resources? There are many hypotheses to answer that question ranging from brand recognition to link placement. It will be important to understand why ADL registered members go to PubMed so frequently in order to better design the ADL site to maximize the success of a user's search experience.

There are two things that stand out about the guest users. They access nursing resources at a higher rate than the rest of the user groups and they access AHEConnect, a portal for accessing online CE courses, and the Online CE calendar at a higher rate than the rest of the user groups. It is difficult to draw a hypothesis about why this might be the case. There might be a connection between these usage patterns and the online Nursing Management Institute. The Nursing Management Institute is a 12 course program that can lead to a certification for nurses wanting to become nurse managers. ADL resources are offered to course participants and are accessible and authenticated directly through the Nursing Management Institute Course pages. This program started as a grant funded project and the ADL resources were given for free to course participants. If this is the case then further study will need to be conducted to ensure that those taking AHEConnect courses are getting connected to the library resource affiliated with their course when a particular course has specific resources assigned.

Institutional affiliates are the second highest user group accessing free resources through the ADL. This follows a logical statistical progression based on the fact that the institutional users are the largest user group within the ADL user database. It would be useful for collection development and site design to gain a better understanding of the institutional users' use of the free resources. Are the institutional users linking out to the free resources with such a high frequency merely due to mathematical strength or is there something about that group of users in particular that leads them to the free resources? Of particular note with the institutional affiliates is the frequency with which they select free patient education materials. If patient education materials are a high demand item, it would helpful to determine the usability of the free materials being accessed versus purchasing licensed patient education products.

AHEC faculty/staff go to free EBM resources for 9% of all of their free resource link outs and they link out to the EBM Center of Excellence for 40% of all of the link outs to that resource. The question is why do AHEC faculty/staff utilize free EBM resources at a higher frequency than other groups? It would be interesting to see if there is some characteristic that can be identified about AHEC faculty/staff that might be used to encourage other groups to increase their use of EBM resources.

The use of free resources and the preponderance of link outs to dermatology resources by the paid individual members can only be analyzed after further study. Is it just one user with a high frequency of use or is this a trend spread out amongst several users? Are the licensed dermatology resources being used at the same frequency rate as the free dermatology resources? A further look at usage of free resources by individual paid members is needed before drawing any conclusions.

This report provides a good starting point in examining the use of free resources. As stated above there are certainly more issues to be examined. An important question is obviously, whether certain resources should be maintained in the collection due to lack of use. Given that it may be that some of the resources would or could be used more if users were made aware of them, this involves additional questions about training and familiarity. In the short term, resources should not be dropped from the collection only due to lack of use, but lack of use can be used as one of several factors in determining whether to allow a resource to remain in the collection.

Finally, in analyzing use of free resources, it is important to note the overall limitations of this survey. This study pulled data from a 6 month time period. Additional resources might be accessed over a longer time frame. While link outs are excellent indicators of frequency of use they do not provide any information about the ultimate usefulness of the resource being accessed. Further studies would need to examine not only the quantitative use of resources, but also their quality. There is no limit to the number of free resources that could be linked through the ADL, but it is important to ensure that the resources linked to are useful to the ADL's target audience. Additional qualitative studies of the utility of the free resources (for example, repeat usage of resource by individuals) could also be conducted to follow-up on the numbers and analysis of this report.

## Abbreviations

**ADL **– The North Carolina AHEC Digital Library

**AHEC **– Area Health Education Centers

**CE **– Continuing Education

**EBM **– Evidence Based Medicine

**LISA **– Library and Information Science Abstracts

**NC **– North Carolina

**UNC **– University of North Carolina at Chapel Hill

## Competing interests

The author(s) declare that they have no competing interests.
